# Chemical and Functional Characteristics of Strawberry Tree (*Arbutus unedo* L.) Honey from Western Greece

**DOI:** 10.3390/foods14091473

**Published:** 2025-04-23

**Authors:** Chrysoula Tananaki, Dimitrios Kanelis, Vasilios Liolios, Maria Anna Rodopoulou, Fotini Papadopoulou

**Affiliations:** Laboratory of Apiculture-Sericulture, Aristotle University of Thessaloniki, Aristotle Farm, 57001 Thermi, Greece; tananaki@agro.auth.gr (C.T.); vliolios@agro.auth.gr (V.L.); rodopoum@agro.auth.gr (M.A.R.); foteinpi@agro.auth.gr (F.P.)

**Keywords:** strawberry tree honey, physicochemical characteristics, total phenolic content, total antioxidant activity, carbohydrate profile, phenolic compounds profile

## Abstract

Strawberry tree honey (*Arbutus unedo* L.) is a rare monofloral honey type with unique characteristics, mainly produced in the Mediterranean region. Despite its distinct qualities, limited research on its physicochemical and biological properties, coupled with the absence of specific legislative standards, hinder its market potential. For this reason, in the present study, we analyzed strawberry tree honey samples collected from beekeepers in Western Greece, focusing on physicochemical properties (moisture, electrical conductivity, HMF, diastase activity, color, pH, acidity), total phenolic content, antioxidant activity, carbohydrate composition, and phenolic compounds profile. The results revealed high moisture content (19.2 ± 1.9%) and electrical conductivity (0.784 ± 0.132 mS cm^−1^), low diastase activity (9.6 ± 3.8 DN), and a strong crystallization tendency (1.01). Additionally, the honey exhibited elevated levels of total phenolic content (1169.9 ± 323.8 mg GAE kg^−1^ honey) and total antioxidant activity (10.98 ± 2.42 mmol Fe^2+^ kg^−1^ honey), compared to other blossom honeys, with homogentisic acid emerging as the dominant phenolic compound. These findings highlight the potential of strawberry tree honey as a high-value product, contributing to its enhanced market promotion.

## 1. Introduction

Nowadays, honey is considered a basic part in consumers’ diets as they embrace healthier lifestyles. The biological properties of honey, such as antioxidant and antimicrobial activities, as well as its sensory characteristics (e.g., color, taste, texture) have been found to depend mainly on its botanical origin, leading to an increase in the demand of monofloral honeys. Combined with the limited geographical areas and short flowering periods required for their production, monofloral honeys typically command higher prices compared to blends. For these reasons, beekeepers around the world tend to produce monofloral honeys (e.g., thyme honey, pine honey, etc.), many of which stand out for their unique characteristics. Furthermore, to compete with low-cost imported honey, beekeepers are increasingly focusing on producing high-quality monofloral honey types to meet market demands.

Strawberry tree (*Arbutus unedo*) honey, a type of honey produced along the Mediterranean, known for its characteristic bitter taste and persistent aroma, has seen increasing demand and consumption in recent years [1,2]. Regardless of its great value, there is limited research on its nutritional and biological benefits, as well as its unique organoleptic properties, which prevents it from receiving the recognition it truly deserves. This type of honey has been mainly explored in Sardinia [2,3,4,5,6], while few papers exist in the literature concerning its pollen profile [1,7], physicochemical characterization [6,7], and biological properties [6,8,9,10,11].

Several researchers have focused on the distinctive phenolic and volatile compounds present in strawberry tree honey. Regarding phenolic compounds, homogentisic acid has been widely reported as a floral origin marker [1,9,12], while Tuberoso et al. [1] additionally identified (±)-2-cis,4-trans-abscisic acid (c,t-ABA), (±)-2-trans,4-trans-abscisic acid (t,t-ABA), and unedone (2-(1,2-dihydroxypropyl)-4,4,8-trimethyl-1-oxaspiro [2.5] oct-7-en-6-one) as significant components, with unedone also confirmed by Montoro et al. [13]. Concerning volatile compounds, Bianchi et al. [3] highlighted α-isophorone, β-isophorone, and 4-oxoisophorone as markers of strawberry tree honey, while Osés et al. [10] proposed 2,6,6-trimethyl-4-oxo-2-cyclohexen-1-carboxaldehyde, 3,4,5-trimethylphenol, and 2-hydroxycyclopent-2-en-1-one as potential markers.

Greece’s diverse climate supports the production of various monofloral honey types, including strawberry tree honey, especially in the western regions; however, it is not produced by many beekeepers. Furthermore, consumer unawareness and the absence of legislative criteria hinder the market promotion of this type of honey, leaving significant quantities unsold. Considering all the above, in the present study, monofloral strawberry tree honey samples were collected from beekeepers in Western Greece and analyzed for their physicochemical and biological properties with the aim to highlight the unique quality characteristics of strawberry tree honey and to further emphasize its nutritional significance. The findings could potentially contribute to the development of legislative standards, helping to define and elevate the quality of strawberry tree honey.

## 2. Materials and Methods

### 2.1. Collection of Strawberry Tree Honey Samples

Strawberry tree honey samples were collected from collaborated beekeepers located in Western Greece (Regional Units of Ioannina, Arta, Thesprotia). Beekeepers were provided with instructions to apply the appropriate beekeeping practices in order to collect representative honey samples. The samples, after their collection, were sent to the Laboratory of Apiculture, Sericulture of Aristotle University of Thessaloniki (AUTH) and kept in a freezer (−18 °C) until their analysis. To certify their botanical origin, the samples were at first studied for their pollen and sensory characteristics. The qualitative pollen analysis was performed using the method established by the International Commission of Bee Botany described by Von der Ohe et al. [14]. Furthermore, a panel of 6 experts tested the honey samples and confirmed their botanical origin, by approving or disapproving the monofloral nature of each sample with a yes or no response, based on its color, taste, odor, and aroma [15]. In total, 37 monofloral strawberry tree honey samples were analyzed.

### 2.2. Analyses of Physicochemical Characteristics

The physicochemical analyses of the collected strawberry tree honey samples were conducted following the methods outlined by Bogdanov et al. [16].

The water content was analyzed using an ATAGO refractometer (ATAGO Co., Ltd., Tokyo, Japan, HHR-2N), while for the determination of the electrical conductivity, an amount calculated using the formula m = 500/(100 − water content (%)) was diluted with deionized water to reach a total volume of 25 mL. The WTW conductometer (Cond315i, WTW GmbH, Weilheim, Germany) was used, the electrode of which was immersed in the solution, providing the value adjusted to 20 °C. The hydroxymethylfurfural (HMF) content was identified by measuring its UV absorbance at 284 nm, and subtracting the background absorbance at 336 nm. Diastase activity was measured through the spectrophotometric method, using a buffered solution of soluble starch and honey incubated in a thermostatic bath at 40 °C.

The pH of the honey solutions was identified using a Nahita 902/4 pH meter (Auxilab S.L., Beriáin, Spain), while for the determination of free acidity, the solutions were titrated with a solution of 0.05 mol L^−1^ sodium hydroxide (NaOH, Lach-Ner, Neratovice, Czech Republic), until reaching the pH value of 8.3.

The color was determined using a honey color analyzer (Hanna, HI-83221, Nușfalău, Romania) in Pfund scale and a Konica Minolta colorimeter (Konica Minolta, CR-410, Tokyo, Japan). To measure the CIE L* a* b* color coordinates, the colorimeter was calibrated using a white standard plate with color coordinates of Υ = 85.8, x = 0.3192, and y = 0.3369. The L* coordinate indicates brightness, ranging from L* = 0 (black) to L* = 100 (colorless). The a* coordinate represents the green/red spectrum, with a* > 0 indicating red and a* < 0 indicating green. The b* coordinate reflects the blue/yellow spectrum, where b* > 0 signifies yellow and b* < 0 signifies blue.

### 2.3. Evaluation of Biological Properties In Vitro

The samples were also analyzed for their antioxidant potential by measuring their total phenolic content (TPC) (Meda et al., 2005) [17] and total antioxidant activity through the Ferric Reducing Power (FRAP) Assay (Benzie and Strain, 1999) [18].

For the determination of TPC, the Folin–Ciocalteu colorimetric method was applied, where honey samples diluted in distilled water (1:10) were filtered and 500 μL of the resulting solution was mixed with 2.5 mL of 0.2 N Folin–Ciocalteu phenol reagent (2 N) (Merck, Darmstadt, Germany) and kept for 5 min at room temperature. Then, 2 mL of 75 g L^−1^ sodium carbonate (Na_2_CO_3_) (≥99.5%, Merck, Darmstadt, Germany) was added and the mixture was incubated for 2 h in the dark at room temperature. The absorbance was measured at 760 nm (Genesys 10S UV-Vis, Thermo Fisher Scientific, Waltham, MA, USA) using a methanol blank as a reference (≥99.8%, Chem-Lab, Zedelgem, Belgium). The analysis was performed in duplicate. For the calibration curve, standard solutions of gallic acid (10–400 mg L^−1^) were used, while the results were expressed as mg gallic acid equivalents (GAEs) per 100 g^−1^ honey.

For the assessment of total antioxidant activity, the honey samples were diluted with distilled water in a (1:5) (*w*/*v*) ratio, which was stirred for 20 min. Then, 3 mL from the FRAP solution was mixed with 100 μL of the honey solution. The fresh FRAP reagent contained a solution of 300 mM CH_3_COONa −CH_3_COOH (≥99%, Chem-Lab, Zedelgem, Belgium) buffer (pH = 3.6), a solution of 10 mM TPTZ (2,4,6-tripyridyl-s-triazine) (≥98.0%, Merck, Darmstadt, Germany) in 40 mM HCl (3 M, Chem-Lab, Zedelgem, Belgium), and an aquatic solution of 20 mM FeCl_3_ × 6H_2_O (>99%, Chem-Lab, Zedelgem, Belgium), in a ratio of 10:1:1. The samples were stirred and transferred to a water bath at 37 °C for 4 min. Absorbance was measured at 593 nm, using water (100 µL) with 3 mL of FRAP as the reference solution. The analysis was performed in duplicate. Standard solutions of iron (II) sulfate heptahydrate (concentration: 50–2000 μΜ) (>99.7%, Chem-Lab, Zedelgem, Belgium) were used for the calibration curves, while the results were expressed as millimoles of iron (II) sulfate heptahydrate per kg of honey (mmol Fe^2+^ kg^−1^ honey).

### 2.4. Carbohydrate and Phenolic Compounds Profile Analysis

The method of Bogdanov et al. [19] was used in order to analyze the carbohydrate profile using high-performance liquid chromatography (HPLC) equipped with a refractive index detector system (RID) (Agilent, 1200 series, Santa-Clara, CA, USA). An amount of 5 g of honey samples was dissolved in a methanol:water (25:75, *v*/*v*) (HPLC grade, ≥99.9%, Chem-Lab, Zedelgem, Belgium) solution to a final volume of 50 mL and filtered through a 0.45 μm disposable syringe filter prior to injection. Carbohydrate separation was conducted using two Zorbax Carbohydrate Analysis columns (4.6 mm ID × 150 mm × 5 μm) (Agilent Technologies, Inc. Headquarters, Santa-Clara, CA, USA) arranged in series and protected by a guard column (NH_2_ Guard Cartridge, 4.6 mm × 12.5 m) (Agilent Technologies, Inc. Headquarters, Santa-Clara, CA, USA). The mobile phase consisted of an acetonitrile:water (75:25, *v*/*v*) (HPLC grade, ≥99.9%, Chem-Lab, Zedelgem, Belgium) solution at a flow rate of 1.8 mL min^−1^. The column and detector were maintained at a constant temperature of 35 °C, with an injection volume of 10 μL. Quantification was performed using five-point calibration curves for each carbohydrate compound. Sixteen carbohydrate compounds were studied: D(−)-fructose, D-(+)-glucose, D(+)-sucrose, D-maltose monohydrate, D-(+)-turanose, D-(+)-trehalose dehydrate, isomaltose, D(+)-maltotriose, D-(+)-melezitose hydrate, erlose, D-raffinose pentahydrate, melibiose, D-panose, maltulose, maltotetraose and isomaltotriose, all of HPLC grade (Merck, Darmstadt, Germany).

To determine the phenolic compounds profile, the methods of Can et al. [20] and Akuyz et al. [21] were followed, with some modifications. Phenolic acids homogentisic, protocatechuic, caffeic, syringic, p-coumaric, ellagic, as well as flavonoids catechin, epicatechin, rutin, quercitrin, quercetin, chrysin (≥98%, Merck, Darmstadt, Germany) were analyzed using a high liquid chromatography with a diode-array detector (HPLC-DAD) (Agilent Technologies, Inc. Headquarters, Santa-Clara, CA, USA). The honey samples (15 g) were mixed with 50 mL of HPLC-grade methanol with continuous stirring until fully dissolved. The solution was filtered and transferred to 50 mL volumetric flasks, where the volume was adjusted with methanol. The prepared solutions were transferred into beakers, sealed with parafilm, and stored at −80 °C for 24 h. After freezing, the samples were lyophilized and subjected to liquid−liquid extraction. Specifically, 20 mL of ultrapure water (Merck, Darmstadt, Germany) and 20 mL of diethyl ether (≥99%, Chem-Lab, Zedelgem, Belgium) were added to each sample. The mixtures were left at room temperature until two distinct layers formed: a diethyl ether phase (up) and a honey−water phase (down). The ether phase was carefully collected into a beaker, stored again at −80 °C for 24 h, and lyophilized a second time. The remaining extract was dissolved in methanol and transferred to HPLC vials, to which 70 μL of internal standard solution of propylparaben (100 ppm) (Extrasynthese, Lyon, France) was added. The final volume in each vial was adjusted to 1 mL.

The samples were analyzed in HPLC-DAD using a C18 column (4.6 × 150 mm, 3 μm) on an Agilent 1200 liquid chromatography system (Agilent Technologies, Inc. Headquarters, Santa-Clara, CA, USA) with a gradient elution program. Two solvent systems were employed: Solvent A (80% acetonitrile in methanol) and Solvent B (2% acetic acid in ultrapure water). The elution progressed from high polarity and low pH to low polarity and high pH. The gradient program was the following: 0–2 min, 95% B; 2–8 min, 95–90% B; 8–11 min, 90–85% B; 11–13 min, 85–75% B; 13–17 min, 75–70% B; 17–30 min, 70–65% B; 30–33 min, 65–0% B; 33–38 min, 0–0% B; 38–40 min, 95% B; and 40–48 min, 95% B. The injection volume was set to 50 μL, with a column temperature maintained at 30 °C. The flow rate was 1 mL min^−1^, and detection was carried out at a wavelength of 290 nm. All solvents were of HPLC grade. Quantification was performed using five-point calibration curves for each phenolic compound.

### 2.5. Statistical Analysis

The statistical analysis was performed in SPSS v.24.0 software (Chicago, IL, USA) and expressed as mean values ± standard deviations. The level of significance was set at a = 0.05.

## 3. Results and Discussion

### 3.1. Physicochemical Characteristics

The botanical origin of the collected samples was at first confirmed by the combination of melissopalynological and sensory analyses. Strawberry tree honey is normally under-represented, while there are no legislative criteria established regarding its pollen percentage. Also, the coexistence of overrepresented pollens may cause a wide variation in the percentages of *Arbutus* pollen [1]. The range of *Arbutus* pollen grains was 4–59%. It should be noted that pollen grains of *Hedera helix* were also found in the samples, with percentages ranging from 31 to 84%. Pollen grains from *Asparagus* sp., *Erica manipuliflora*, Fabaceae, and *Trifolium* sp. were also detected, while regarding nectarless plants, pollen grains from *Quercus* sp. and Cyclamen type were mostly found. Persano Oddo et al. [7] pointed out higher limits for Arbutus pollen grains (>8%), which could be attributed to the different geographical origin of the specific honey type.

After the confirmation of their botanical origin, the samples were analyzed for their physicochemical characteristics. The results are given in Table 1.

**Figure 1:**
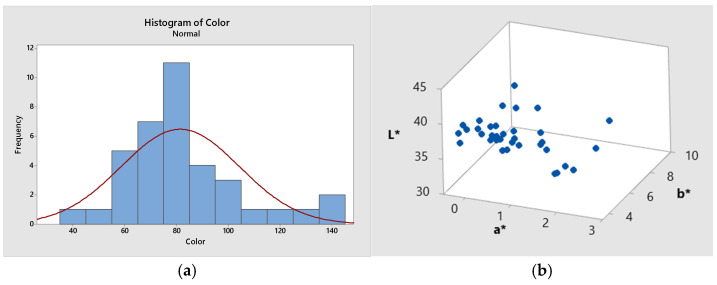
Color (mm Pfund) (**a**) and L*, a*, b* parameters (**b**) in strawberry tree honey samples (*n* = 37). L* represents the clarity (L* = 0 black and L* = 100 colorless), a* the green/red color component (a* > 0 red, a* < 0 green), and b* the blue/yellow color component (b* > 0 yellow, b* < 0 blue).

The collected samples exhibited a relatively high mean moisture content (19.2 ± 1.9%), with 38% exceeding the moisture limit (lower than 20%) established by the European legislation [22]. Electrical conductivity was also found in high levels (mean value: 0.784 ± 0.132 mS cm^−1^), with 49% of the samples surpassing the threshold of 0.8 mS cm^−1^ which distinguishes blossom from honeydew honeys; however, this particular honey type is classified as an exception under the Honey Directive [22], regarding its conductivity. Additionally, acidity was notably high (average: 34.1 ± 7.6 meq kg^−1^), while 30% of the samples exceeded 40 meq kg^−1^, leading to restrains of their shelf life. Acidity is a useful criterion for the evaluation of fermentation and characterization of monofloral honeys, and according to the Honey Directive [22], it should be lower than 50 meq kg^−1^. Regarding pH, all the samples were acidic, with a mean pH value of 4.03 ± 0.16. Moreover, the diastase activity was found to be relatively low (mean value: 9.6 ± 3.8 DN), and 38% of the samples had diastase values lower than 8 DN, suggesting strawberry tree honey to be added to the legislative exceptions [23], considering that the minimum legislative value of diastase activity is 8 DN, with exceptions including honeys with low enzyme content that require diastase activity above 3 DN and HMF content below 15 mg kg^−1^ (e.g., citrus honeys) [22]. The HMF content was below 15 mg kg^−1^ (3.1 ± 3.3 mg kg^−1^), proving that the samples were fresh and unprocessed. Finally, the honey was classified as light brown on the Pfund scale, with an average value of 81.4 ± 22.8 mm Pfund, while most samples had color values between 60 and 80 mm Pfund (Figure 1a).

This classification aligns with the L* mean value of 36.80 ± 1.27, indicating predominantly yellow hues followed by green tones, as reflected in the b* and a* mean values (5.22 ± 1.09 and 0.66 ± 0.77, respectively), and as presented in Figure 1b. The yellow hues are mainly attributed to the presence of carotenoids and flavonoids in the nectar, while the green hues may be due to a high chlorophyll content [24].

The findings of the present study coincided with those reported by Persano Oddo et al. [7] regarding moisture content (18.9 ± 1.9%), electrical conductivity (0.740 ± 0.07 mS cm^−1^), HMF levels (4.4 ± 3.2 mg kg^−1^), and pH (4.2 ± 0.1) and by Rodopoulou et al. [25], as well (moisture content: 19.0 ± 1.7%, electrical conductivity: 0.740 ± 0.10 mS cm^−1^, pH: 4.2 ± 0.1). However, Persano Oddo et al. [7] observed lower color values (70 ± 10 mm Pfund), lower diastase activity (5.2 ± 2.8 DN), and slightly higher acidity (39.6 ± 8.3 meq kg^−1^). Castiglioni et al. [26] also found lower color values (70 ± 10 mm Pfund), compared to the results of this study. On the other hand, Petri and Tarola [6], in their analysis of five honey samples from Sicily and five from Sardinia, recorded higher HMF levels (11.1 ± 1.05 mg kg^−1^ in honeys from Sicily and 19.63 ± 4.65 mg kg^−1^ in honeys from Sardinia) but similar pH values (4.26 ± 0.10 and 4.24 ± 0.10) to those found in this study, while the acidity levels observed in the present research were more consistent with the honey samples from Sardinia (30.81 ± 3.63 meq kg^−1^) than with those from Sicily (39.72 ± 3.11 meq kg^−1^).

### 3.2. Antioxidant Properties

**Figure 2:**
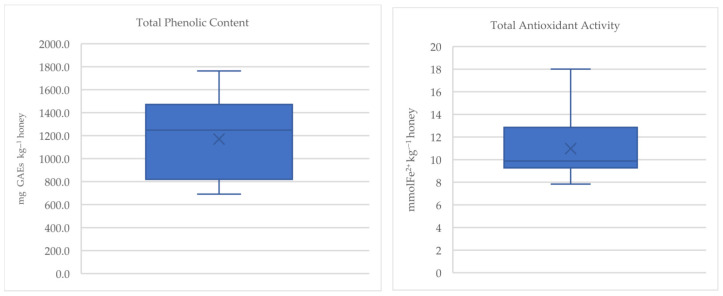
Total phenolic content and total antioxidant activity of strawberry tree honey.

The collected honey samples were also analyzed for their total phenolic content (TPC) and their total antioxidant activity by applying the FRAP assay (Figure 2).

The TPC values were relatively high, averaging 1169.9 ± 323.8 mg GAEs kg^−1^ honey. Petri and Tarola [6] found similar values (1035.3 mg GAEs kg^−1^ honey for honey from Sicily, 956.4 mg GAEs kg^−1^ honey for honey from Sardinia) along with Rosa et al. [8] (972 mg GAEs kg^−1^ honey), while Osés et al. [10] reported slightly higher values (1500 ± 300 mg GAEs kg^−1^ honey). Afrin et al. [4] observed lower total phenolic values (from 690 ± 100 mg GAEs kg^−1^ honey to 1000 ± 200 mg GAE kg^−1^ honey), and Castiglioni et al. [26] as well (850 ± 133 mg GAEs kg^−1^ honey). Furthermore, the mean value of total antioxidant activity was 10.98 ± 2.42 mmol Fe^2+^ kg^−1^ honey, similar to the values found by Rosa et al. [8] (11.7 ± 1.7 mmol Fe^2+^ kg^−1^ honey) and Tuberoso et al. [27] (12.0 ± 2.2 mmol Fe^2+^ kg^−1^ honey), while it was higher than that identified by Afrin et al. [4] (5.1–9.2 mmol Fe^2+^ kg^−1^ honey). In comparative studies, strawberry tree honey was found among the types with the highest antioxidant activity, attributing this potential to its amount of polyphenols [9,26].

### 3.3. Carbohydrate and Phenolic Compounds Profile

**Figure 3:**
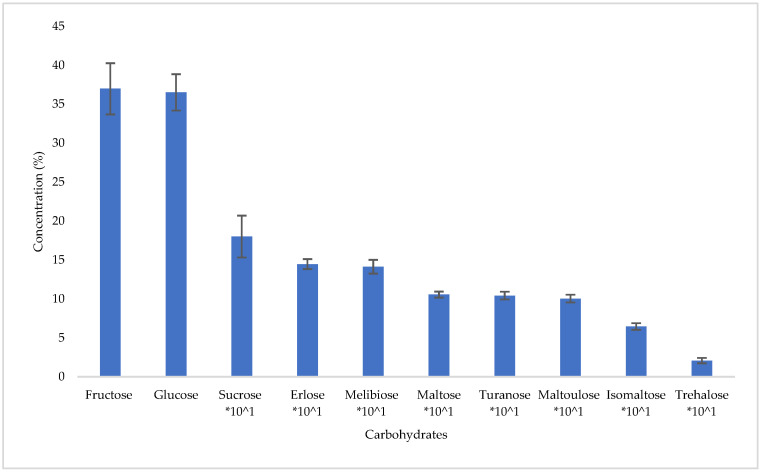
Carbohydrates mean concentration (%) in strawberry tree honey.

Carbohydrate profiling revealed an average fructose content of 36.99 ± 3.29% and glucose at 36.53 ± 2.34% (Figure 3), the sum of which (73.52%) is above 60%, the lower limit for blossom honeys [22].

The sum of the main sugars fructose and glucose, as well as their ratio, have been found to affect the degree of crystallization in honey; honeys with a low fructose/glucose ratio (<1.11) tend to crystallize faster compared to honeys with a high fructose/glucose ratio [28]. In the present study, strawberry tree honey had a low fructose/glucose ratio (1.01), showing a fast rate of crystallization; therefore, care should be taken during its shelf storage. Additionally, sucrose levels were generally low; however, 22% of the samples exceeded the legislative limit of 5% [22]. This could be due to the late autumn collection of strawberry tree honey, which limits the time available for honey bees to process the nectar.

Erlose, melibiose maltose, turanose, and maltulose followed in lower percentages (average: 1.45 ± 0.64%, 1.41 ± 0.88%, 1.06 ± 0.39%, 1.04 ± 0.50%, and 1.00 ± 0.50%, respectively), while even lower mean concentrations were observed for isomaltose (0.65 ± 0.42%) and trehalose (0.21 ± 0.34%). From the carbohydrates analyzed, raffinose, maltotriose, panose, isomaltotriose, and maltotetraose were not detected. The results are in agreement with those presented by Persano Oddo et al. [7],who also found fructose (37.2 ± 2.4%), glucose (32.1 ± 1.1%), sucrose (1.5 ± 0.9%), maltose (1.2 ± 0.5%), isomaltose (0.8 ± 0.3%), and erlose.

**Figure 4:**
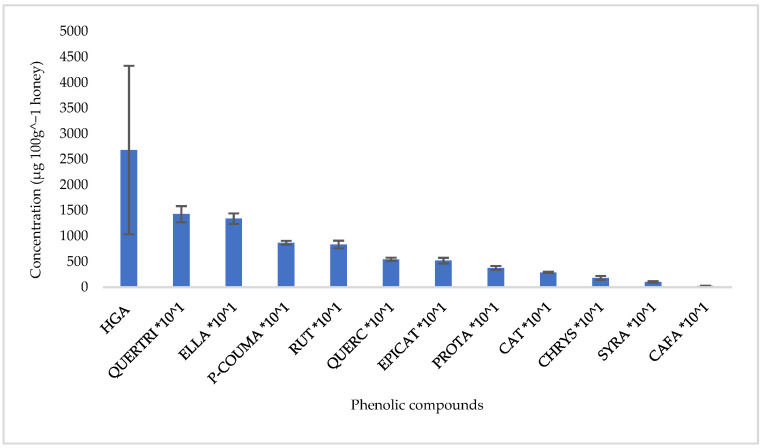
Phenolic compounds concentration in Arbutus honey samples from Western Greece (HGA: homogentisic acid; QUERTRI: quercitrin; ELLA: ellagic acid; p-COUMA: p-coumaric acid; RUT: rutin; QUERC: quercetin; EPICAT: epicatechin; PROTA: protocatechuic acid; CAT: catechin; CHRYS: chrysin; SYRA: syringic acid; CAFA: caffeic acid).

Regarding phenolic compounds, the homogentisic acid was detected in higher concentrations (2681.1 ± 1645.5 μg 100 g^−1^ honey) (Figure 4), in agreement with other studies, proposing the specific compound as a potential origin marker [8,12].

Rosa et al. [8] even reported that the homogentisic acid represented 50–60% of the total phenolic compounds in the honey, while they found that the phenolic compound itself showed a high antioxidant activity and a protective effect against the oxidation of LDL cholesterol, which makes an important contribution to the high antioxidant and antiradical properties of strawberry tree honey. Homogentisic acid has been also found to offer protection against light and oxidation stress, ROS and DPPH radical scavenging activities, and protecting human peripheral blood lymphocytes against irinotecan-induced cytogenetic damage [29]. The compounds ellagic acid, quercitrin, p-coumaric acid, and rutin followed in mean concentrations 134.15 ± 155.56 μg 100 g^−1^ honey, 134.10 ± 103.28 μg 100 g^−1^ honey, 86.82 ± 38.46 μg 100 g^−1^ honey, and 83.53 ± 73.68 μg 100 g^−1^ honey, respectively. The compounds caffeic acid, p-coumaric acid, syringic acid, ellagic acid, rutin, and chrysin are also noted in the study by Petri and Tarrola [6], while caffeic acid, p-coumaric acid, quercetin, and chrysin were detected in the study of Jurič et al. [30]. The comparison of the studied phenolic compounds in monofloral honey types with the results of other studies is difficult, not only due to the origin of the honey, but also due to the process applied during the extraction of phenolic compounds and their analysis. The results between studies can be comparable only when the same analysis method has been followed [31]. As for the phenolic compounds ellagic acid, quercitrin, p-coumaric acid, and rutin, their biological potential has been researched, confirming their role as antioxidant, antimicrobial, anti-allergic, anti-inflammatory, and anti-apoptotic agents [32,33,34,35].

The study focuses on strawberry tree honey, a unique and rare monofloral honey type, which has not been extensively researched compared to the more common honey types. The researches that exist so far mostly refer to the region of Italy-Sardinia. To our knowledge, this is the first time that a comprehensive study, including a large number of samples (*n* = 37) and the examination of different parameters (water content, electrical conductivity, HMF, diastase activity, color, pH, acidity, total phenolic content, total antioxidant activity, carbohydrate profile, phenolic compounds profile) was being conducted on Arbutus honey produced in Western Greece, providing data that could help in promoting regional honey production and supporting local beekeepers. The large number of samples aids in documenting deviations in certain physicochemical characteristics, which support the improvement in legislation regarding this type of honey. Moreover, most of the researches identified the main carbohydrates (fructose and glucose), while in the present study, besides the main carbohydrates, disaccharides and trisaccharides were also determined: D(+)-sucrose, D-maltose monohydrate, D-(+)-turanose, D-(+)-trehalose dehydrate, isomaltose, D(+)-maltotriose, D-(+)-melezitose hydrate, erlose, D-raffinose pentahydrate, melibiose, D-panose, maltulose, maltotetraose, and isomaltotriose, focusing on the identification of a carbohydrate profile, instead of specific carbohydrates. Finally, few studies exist that have detected specific phenolic compounds, besides the homogentisic acid, the results of which could be used in future clinical tests.

## 4. Conclusions

As consumer preferences increasingly shift toward distinctive food products, the results of the present study could support the promotion of strawberry tree honey, a monofloral honey type with distinct organoleptic characteristics. It was found that the specific honey type has light brown color, and is characterized by high moisture content and electrical conductivity, low diastase activity, and strong tendency to crystallize. It is also rich in phenolic compounds, with homogentisic acid being the most abundant. Significant amounts of the compounds quercitrin, ellagic acid, p-coumaric acid, and rutin were also detected, all of which are known for their beneficial effects on human health. 

## Figures and Tables

**Table 1:** Physicochemical characteristics (mean values, standard deviations, min, max) of strawberry tree honey.

Physicochemical Parameters	Moisture (%)	Electrical Conductivity (mS cm^−1^)	HMF (mg kg^−1^)	Diastase Activity (DN)	pH	Acidity (meq kg^−1^)
Mean value ± Standard deviation	19.2 ± 1.9	0.784 ± 0.132	3.1 ± 3.3	9.6 ± 3.8	4.3 ± 0.16	34.1 ± 7.6
Min-Max	16.4–24.0	0.454–1.120	0.0–12.0	3.2–18.0	3.72–4.41	17.5–49.0

## Data Availability

The original contributions presented in the study are included in the article, further inquiries can be directed to the corresponding author. Data supporting the reported results are stored at the Laboratory of Apiculture-Sericulture, AUTH.
